# Oxidative Stress-Related DNA Damage in Patients with Idiopathic Granulomatous Mastitis: A Prospective Case–Control Study

**DOI:** 10.3390/jcm15114228

**Published:** 2026-05-30

**Authors:** Ceren Gonultas, Adem Akcakaya, Abdurrahim Kocyigit, Gulnihal Sisman, Berrin Papila, Mehmet Velidedeoglu, Hasan Dagmura

**Affiliations:** 1 Department of General Surgery, Dogubayazit Dr. Yasar Eryilmaz State Hospital, Ministry of Health, 04400 Agri, Turkey; 2 Department of General Surgery, Bezmialem Vakif University, 34093 Istanbul, Turkey; aakcakaya@bezmialem.edu.tr (A.A.); hasan.dagmura@bezmialem.edu.tr (H.D.); 3 Department of Medical Biochemistry, Bezmialem Vakif University, 34093 Istanbul, Turkey; akocyigit@bezmialem.edu.tr (A.K.); gulnihalsisman@gmail.com (G.S.); 4 Department of General Surgery, Istanbul University-Cerrahpasa, 34093 Istanbul, Turkey; berrin.papila@iuc.edu.tr (B.P.); mehmet.velidedeoglu@iuc.edu.tr (M.V.)

**Keywords:** idiopathic granulomatous mastitis, oxidative stress, DNA damage

## Abstract

**Background/Objectives**: Idiopathic granulomatous mastitis (IGM) is a rare, benign, chronic inflammatory disease of the breast that may present with recurrent and treatment-resistant courses and can clinically and radiologically mimic breast cancer. Despite its benign nature, IGM may significantly impair quality of life, and its underlying pathophysiology remains unclear. This study aimed to evaluate oxidative stress and DNA damage in patients with IGM. **Methods**: In this prospective case–control study, 28 patients with clinically and histopathologically confirmed idiopathic granulomatous mastitis who had not received corticosteroid or immunosuppressive therapy within the previous six months were enrolled. An age-matched control group of 27 healthy women was included. Venous blood and urine samples were collected for the assessment of total oxidant status (TOS), total antioxidant status (TAS), and calculation of the oxidative stress index (OSI). Mononuclear leukocyte DNA damage was evaluated using the alkaline Comet assay, and urinary 8-hydroxy-2′-deoxyguanosine (8-OHdG) levels were measured by ELISA. Sociodemographic data, laboratory and imaging results of the patients were also evaluated. **Results**: The mean ages of the patient and control groups were 37.3 ± 5.3 and 35.4 ± 8.6 years, respectively, with no significant difference (*p* = 0.081). Patients exhibited significantly higher inflammatory markers and oxidative stress parameters, including TOS, OSI, and urinary 8-OHdG (*p* < 0.05), whereas TAS did not differ between groups (*p* = 0.534). Comet assay analysis demonstrated significantly increased tail intensity (%) and tail moment in the patient group (*p* = 0.029 and *p* = 0.016). **Conclusions**: IGM is associated with increased oxidative stress and mononuclear leukocyte DNA damage. These findings suggest that oxidative stress-induced DNA damage may play a role in the pathophysiology of IGM and highlight the potential value of antioxidant-based therapeutic strategies as adjunctive treatment options.

## 1. Introduction

Idiopathic granulomatous mastitis (IGM) is a rare, chronic inflammatory disease of the breast with an unknown etiology. It has an annual prevalence of 2.4 in 100,000 and an incidence rate of 0.37%. Although it is a benign disease, IGM may follow a complicated, treatment-resistant course with a tendency for recurrence. The disease was first described by Kessler and Wolloch in 1972, and since then, various etiological hypotheses have been proposed [1,2,3]. Suggested contributing factors include autoimmune mechanisms, pregnancy or breastfeeding history, oral contraceptive use, hyperprolactinemia, smoking, and alpha-1 antitrypsin deficiency, with autoimmune-mediated inflammation considered the most plausible underlying mechanism [4,5]. Clinically and radiologically, IGM may closely mimic breast cancer, making differential diagnosis challenging. Despite being classified as a benign breast disease, its chronic nature and recurrent episodes may significantly impair patients’ quality of life. Clinically, breast mass, tenderness, nipple discharge and retraction, skin ulcerations, abscesses, and sinus or fistula formation can be seen [6,7]. Definitive diagnosis relies on histopathological examination of breast tissue obtained by biopsy, which typically demonstrates a distinctive chronic inflammatory pattern characterized by aggregates of activated macrophages forming non-caseating granulomas [8].

Although no specific ethnic predisposition has been clearly identified, epidemiological data suggest a higher prevalence of IGM in Mediterranean and Asian countries. The increased number of reported cases from developing regions in these areas raises the possibility that genetic susceptibility combined with environmental factors may play a role in disease pathogenesis [9,10]. There is no consensus concerning the definitive treatment approach. Management strategies are individualized based on clinical presentation and range from conservative observation to local or systemic corticosteroids, antibiotics, immunomodulatory therapies, phytotherapy, and, in refractory cases, surgical interventions including mastectomy [11].

Oxidative stress is a major contributor to DNA damage and results from an imbalance between reactive oxygen species (ROS) production and antioxidant defense mechanisms, leading to oxidative injury of cellular macromolecules, including DNA, proteins, and lipids [12,13,14]. Persistent oxidative stress may induce DNA strand breaks, oxidative base modifications, and genomic instability, particularly in chronic inflammatory conditions.

The alkaline single-cell gel electrophoresis technique (Comet assay) is a sensitive and widely used method for evaluating DNA damage at the single-cell level and has broad applications in biomonitoring and genotoxicity studies [15,16]. Another established marker of oxidative DNA damage is urinary 8-hydroxy-2′-deoxyguanosine (8-OHdG), a ROS-induced oxidative DNA product that can be quantified by ELISA and reflects systemic oxidative stress [17].

### Aim

We hypothesized that patients with idiopathic granulomatous mastitis exhibit increased oxidative stress and oxidative stress-related mononuclear leukocyte DNA damage compared with healthy controls. Thus, this study was designed to investigate oxidative stress parameters, urinary 8-OHdG levels as a biomarker of oxidative DNA damage, and DNA damage evaluated using the alkaline comet assay in patients with IGM.

## 2. Materials and Methods

In this prospective study, a total of 28 patients with clinically and histopathologically confirmed granulomatous mastitis, classified as idiopathic due to the absence of an identifiable etiological cause, were included. The study was conducted in accordance with the STROBE reporting guidelines. Patients were conducted from the General Surgery Clinic of Bezmialem Vakıf University Faculty of Medicine Hospital and the Granulomatous Mastitis Outpatient Clinic of İstanbul University–Cerrahpaşa, Cerrahpaşa Faculty of Medicine. All patients had not received any systemic or local corticosteroid or immunosuppressive therapy within the preceding six months. In addition, 27 age-matched healthy female volunteers were enrolled as the control group. The study protocol was approved by the Bezmialem Vakıf University Ethics Committee (16.10.2023-E.126508) and conducted in accordance with the Declaration of Helsinki. After providing detailed verbal information about the study, written informed consent was obtained from all participants in both the patient and control groups.

### 2.1. Inclusion Criteria

The study included female participants aged 18 years or older with histopathologically confirmed idiopathic granulomatous mastitis, diagnosed between 2023 and 2024 at Bezmialem Vakıf University Faculty of Medicine Hospital and İstanbul University–Cerrahpaşa Faculty of Medicine Hospital. Patients were required to have breast biopsy findings consistent with granulomatous mastitis with no identifiable etiological cause and have negative microbiological investigations for tuberculosis and fungal infections and no history of corticosteroid or immunosuppressive therapy within the preceding six months. The control group was composed of age-matched healthy individuals. In addition to age matching, participants were screened for medical history, and those with previous systemic diseases, prior surgical interventions, or any ongoing inflammatory or infectious conditions were excluded to minimize potential confounding factors.

### 2.2. Exclusion Criteria

Exclusion criteria included male sex, pregnancy, lactation, malignancy, specific granulomatous mastitis secondary to infectious or systemic inflammatory diseases, positive tuberculosis or fungal cultures, chronic autoimmune disease requiring active anti-inflammatory treatment, and inadequate clinical or histopathological data.

### 2.3. Blood Sample Collection and Biochemical Analysis

Peripheral venous blood and urine samples were obtained from all participants under sterile conditions during outpatient evaluation. Patients were neither required to fast nor scheduled for sampling at a particular time of day. Approximately 10 mL of venous blood was collected into EDTA-containing tubes (BD Vacutainer®, Becton Dickinson, Franklin Lakes, NJ, USA) and immediately transferred to the laboratory for processing. Before centrifugation, a portion of whole blood (250 μL) was separated and stored at −80 °C for subsequent DNA damage analysis. The remaining samples were centrifuged at 3000 rpm for 10 min, and plasma fractions were aliquoted and preserved at −80 °C until analysis. Urine specimens collected for 8-hydroxy-2′-deoxyguanosine (8-OHdG) measurement were similarly stored at −80 °C.

Oxidative stress parameters, including total oxidant status (TOS) and total antioxidant status (TAS), were analyzed using the automated colorimetric methods described by Erel et al. [13]. TOS results were expressed as μmol H_2_O_2_ equivalent/L, whereas TAS values were expressed as mmol Trolox equivalent/L. The oxidative stress index (OSI), reflecting the balance between oxidant and antioxidant capacity, was calculated using the following formula:

OSI = [TOS (μmol H_2_O_2_ Eq/L)/TAS (mmol Trolox Eq/L)] × 100

### 2.4. Assessment of DNA Damage by the Comet Assay

Mononuclear leukocyte DNA damage was evaluated using a modified alkaline single-cell gel electrophoresis (Comet assay) protocol based on the method originally described by Singh et al. [16]. Frozen blood samples were rapidly thawed at 37 °C and mixed with low-melting-point agarose before being layered onto microscope slides precoated with normal-melting-point agarose. Following solidification, the slides were incubated in cold lysis solution to remove cellular membranes and facilitate DNA unwinding.

The slides were subsequently exposed to alkaline electrophoresis buffer for DNA unwinding and electrophoresis under standardized conditions. After neutralization and fixation, DNA was stained with ethidium bromide and visualized using a fluorescence microscope (Leica DM100, Wetzlar, Germany). For each sample, approximately 50 randomly selected cells were analyzed using Comet Assay IV image analysis software Version 4.3 (Perspective Instruments Ltd., Suffolk, UK). DNA damage was quantified using tail intensity (%) and tail moment parameters.

### 2.5. Urinary 8-Hydroxy-2′-Deoxyguanosine (8-OHdG) Measurement

Reactive oxygen species induce various forms of oxidative DNA base damage, among which 8-hydroxy-2′-deoxyguanosine (8-OHdG) is one of the most sensitive and widely used biomarkers. Urinary 8-OHdG levels were measured using a commercial enzyme-linked immunosorbent assay (ELISA) kit, according to the manufacturer’s instructions. Absorbance values were read using an ELISA microplate reader (Thermo Fisher Scientific Inc., Waltham, MA, USA).

#### Statistical Analysis

Descriptive statistics were presented as mean ± standard deviation, median (minimum–maximum), frequency, and percentage, as appropriate. The distribution of variables was assessed using the Kolmogorov–Smirnov and Shapiro–Wilk tests. For comparisons between independent groups, the independent samples *t*-test was used for normally distributed quantitative variables, while the Mann–Whitney U test was applied for non-normally distributed variables. Categorical variables were analyzed using the chi-square test or Fisher’s exact test when chi-square assumptions were not met. Receiver operating characteristic (ROC) curve analysis was performed to determine effect size and optimal cut-off values. Univariate and multivariate logistic regression analyses were used to evaluate effect estimates. Pearson or Spearman correlation analyses were conducted according to data distribution. All statistical analyses were performed using SPSS software version 27.0 (IBM Corp., Armonk, NY, USA).

## 3. Results

All participants included in the study were female. The mean age at diagnosis was 37.3 ± 5.3 years in the patient group and 35.4 ± 8.6 years in the control group, with no significant difference between groups (*p* = 0.081). The age at menarche was significantly lower and the spontaneous abortion rate was significantly higher in the patient group compared with controls (*p* < 0.05). No significant differences were observed between groups regarding parity, age at first birth, number of births, induced abortion rate, menopausal status, smoking, oral contraceptive use, history of breastfeeding, family history of breast cancer, or the presence of autoimmune disease (Table 1).

**Figure 1:**
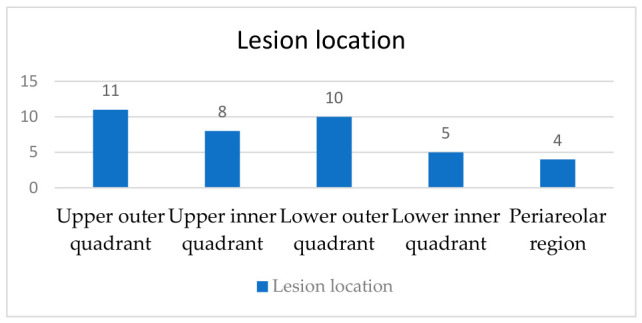
Localization of lesions by breast quadrants.

Breast pain was reported by all patients. Lesions were predominantly unilateral and most frequently located in the upper outer quadrant. (Figure 1) A history of abscess drainage, axillary lymphadenopathy, and fistula formation was common. Previous antibiotic use was reported in 85.7% of patients, while 42.9% had a history of systemic and 35.7% of local corticosteroid use; none had received corticosteroids or immunosuppressive therapy within the preceding six months. Most diagnoses were established by tru-cut biopsy (%78.6) (Table 2).

Laboratory analysis revealed significantly higher white blood cell (WBC) count, neutrophil, lymphocyte counts, neutrophil-to-lymphocyte ratio (NLR), urea, creatinine, lactate dehydrogenase (LDH), and potassium levels in the patient group, while hemoglobin (HB) and hematocrit (HCT) levels were significantly lower (*p* < 0.05). No significant differences were observed for other routine hematological and biochemical parameters. Regarding oxidative stress parameters, TOS, OSI, and urinary 8-OHdG levels were significantly higher in patients compared with controls (*p* < 0.05), whereas TAS did not differ significantly between groups (Table 3).

**Figure 2:**
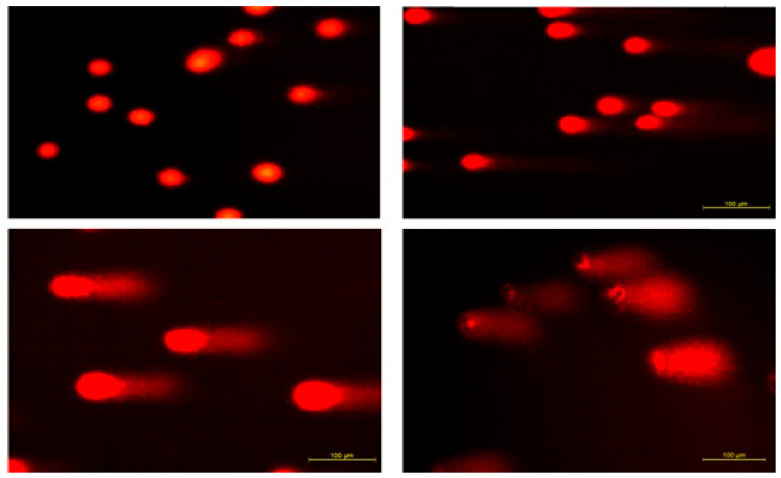
Representative images of damaged DNA exhibiting a comet-like appearance in the patient group, as assessed by the alkaline single-cell gel electrophoresis (Comet assay).

To assess mononuclear leukocyte DNA damage, a modified alkaline single-cell gel electrophoresis (Comet assay) method was employed. Venous blood samples collected in EDTA tubes were aliquoted (250 µL) into separate Eppendorf tubes prior to centrifugation. Due to cell loss during thawing from −80 °C and suspension in low-melting-point agarose, tail intensity and tail moment parameters could be calculated in 14 of 27 patients and 15 of 28 controls. The percentage of tail intensity and tail moment values were significantly higher in the patient group compared with the control group (*p* = 0.029 and *p* = 0.016, respectively) (Table 4) (Figure 2).

### ROC Analysis and Correlations

**Figure 3:**
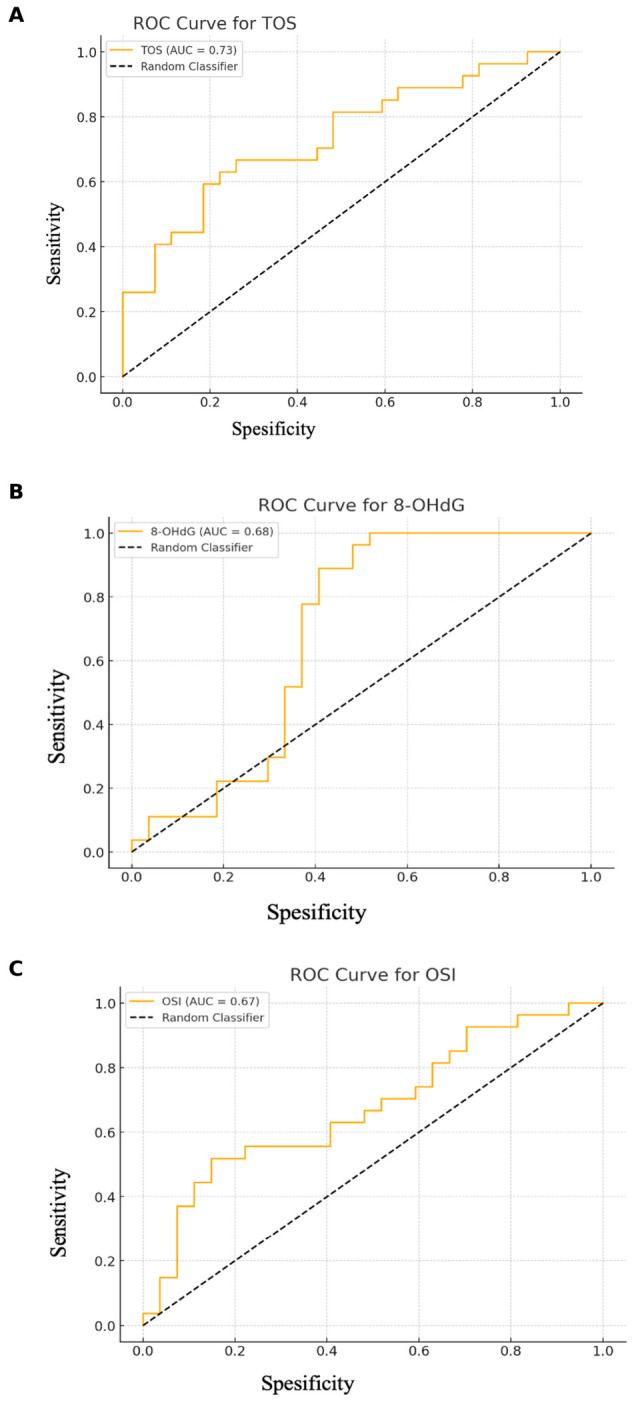
Receiver operating characteristic (ROC) curves of oxidative stress markers for discrimination between patient and control groups. (**A**) Total oxidant status (TOS), (**B**) 8-hydroxy-2′-deoxyguanosine (8-OHdG), and (**C**) oxidative stress index (OSI). The dashed line represents the performance of a random classifier.

ROC curve analysis showed that TOS, urinary 8-OHdG, and OSI each demonstrated significant discriminatory ability in distinguishing patients from controls, with AUC values of 0.73, 0.68, and 0.67, respectively. The optimal cut-off values were 1.36 for TOS (*p* = 0.009), 24.19 for urinary 8-OHdG (*p* = 0.0204), and 178.39 for OSI (*p* = 0.028). At these thresholds, TOS provided 62.96% sensitivity and 77.78% specificity, urinary 8-OHdG showed 100% sensitivity but 48.15% specificity and OSI demonstrated 85.19% specificity with 51.85% sensitivity (Figure 3).

**Figure 4:**
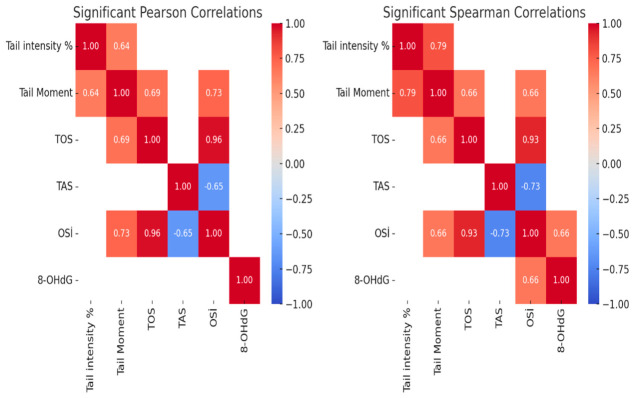
Correlation plots of DNA damage, 8-OHdG, and oxidative stress parameters in the control and patient groups.

Tail intensity (%) showed a moderate positive correlation with tail moment (r = 0.64, *p* = 0.0007), TOS (r = 0.73, *p* < 0.001), and OSI (r = 0.74, *p* < 0.001). In contrast, tail intensity (%) was negatively correlated with TAS (r = −0.51, *p* = 0.005) and showed a moderate positive correlation with urinary 8-OHdG levels (r = 0.45, *p* = 0.015). Tail moment demonstrated significant positive correlations with TOS (r = 0.69, *p* < 0.001), OSI (r = 0.73, *p* = 0.002), and urinary 8-OHdG (r = 0.56, *p* = 0.002), while showing a negative correlation with TAS (r = −0.53, *p* = 0.003). A strong positive correlation was observed between TOS and OSI (r = 0.96, *p* = 0.001). Additionally, TOS was positively correlated with urinary 8-OHdG (r = 0.66, *p* < 0.001) and negatively correlated with TAS (r = −0.53, *p* = 0.003). TAS showed a significant negative correlation with OSI (r = −0.73, *p* = 0.001) and urinary 8-OHdG levels (r = −0.52, *p* = 0.004). Finally, OSI demonstrated a moderate positive correlation with urinary 8-OHdG (r = 0.66, *p* < 0.001). These correlations indicate a strong association between oxidative stress and DNA damage, with antioxidant capacity showing an inverse relationship (Table 5) (Figure 4).

## 4. Discussion

IGM remains a challenging clinical entity due to its ability to mimic breast cancer, the absence of a standardized treatment algorithm, and its unpredictable clinical course, including treatment resistance and recurrence. Despite extensive investigation, the etiopathogenesis of IGM has not been clearly defined. Proposed contributing factors such as hormonal imbalance, autoimmune mechanisms, smoking, infectious agents, and alpha-1 antitrypsin deficiency suggest that dysregulated inflammatory and immune responses may play a central role in disease development [2,5,18]. Currently, increasing emphasis has been placed on the theory that IGM has an autoimmune basis. In the literature, a favorable response to corticosteroid and immunosuppressive therapies, reports of patients presenting with extramammary manifestations such as erythema nodosum or arthritis, and the demonstration of T-lymphocyte predominance in immunohistochemical studies all support the autoimmune hypothesis. Oxidative stress is a well-recognized mediator of chronic inflammation and immune-related tissue injury and has been implicated in the pathogenesis of several granulomatous and autoimmune diseases. Excessive production of reactive oxygen species may overwhelm antioxidant defense mechanisms, leading to cellular damage and genomic instability. A review of the current literature reveals no studies investigating oxidative stress and mononuclear leukocyte DNA damage in patients with IGM [19,20,21,22].

Consistent with large-scale literature, including the systematic review by Martinez-Ramos et al. encompassing 70 studies and 3060 patients, IGM predominantly affects young to middle-aged women in the early postpartum period, and the age distribution observed in our cohort (mean age: 37.3 ± 5.3 years) aligns closely with previously reported data with no statistically significant difference observed between the groups [21]. Oral contraceptives have been suggested as a potential etiological factor in IGM by increasing mammary secretions; however, reported associations in the literature vary widely (0–42%). In our study, oral contraceptive use was observed in 2 patients (7.1%). Smoking has also been proposed as a possible contributing factor, although no definitive association with IGM has been established [23,24]. No statistically significant differences were observed between the patient and control groups with respect to menopausal status, smoking prevalence, oral contraceptive use, or hyperprolactinemia (*p* > 0.05).

Management of idiopathic granulomatous mastitis remains challenging due to heterogeneous study populations, small sample sizes, and the absence of a clearly defined etiology, resulting in a wide spectrum of treatment approaches ranging from clinical observation to radical surgery. Reported treatment options include conservative follow-up, abscess drainage with antibiotics, nonsteroidal anti-inflammatory drugs, and, in refractory or complicated cases, local or systemic corticosteroids, colchicine, methotrexate, azathioprine, prolactin inhibitors, or surgical excision [25,26,27]. Systemic corticosteroids and immunosuppressive agents have demonstrated favorable outcomes in selected patients; however, recurrence or treatment resistance remains common, reported in up to 20–50% of cases [28,29,30]. In our study, most patients had received antibiotics prior to diagnosis (85.7%), while systemic and local corticosteroid use was observed in 42.9% and 35.7% of patients, respectively, consistent with previously published treatment patterns. In addition to these treatment strategies, the potential role of diet in IGM management should also be considered, given the growing evidence supporting autoimmune and inflammatory mechanisms in its pathogenesis. Although the role of diet has not been clearly established in IGM, pro-inflammatory dietary patterns have been associated with increased levels of inflammatory mediators such as interleukin-6 and tumor necrosis factor-α, which may contribute to chronic granulomatous inflammation. Conversely, anti-inflammatory diets rich in fruits, vegetables, vitamin D and omega-3 fatty acids may reduce systemic inflammation and oxidative stress, potentially influencing immune regulation and disease activity [31,32,33].

Immune imbalance plays important roles in the onset and progression of autoimmune diseases. Aberrantly activated immune cells release large amounts of cytokines, triggering autoimmune attacks on host tissues. This overexpression of cytokines further exacerbates immune system hyperactivity, creating a cycle that worsens disease severity. Studies have demonstrated that in IGM, dysregulation of T cells, B cells, and Natural killer (NK) cells, along with elevated release of inflammatory cytokines, are central to the disease process. These immune dysregulations can be alleviated by immunosuppressive treatments, suggesting that immune imbalance is a key mechanism underlying the pathological damage in IGM [34].

Dogan et al. demonstrated that patients with IGM had significantly higher neutrophil and eosinophil counts, an increased neutrophil-to-lymphocyte ratio, and a higher proportion of non-classical monocytes compared with controls (*p* = 0.006, *p* = 0.022, *p* = 0.008, and *p* = 0.000, respectively), while CD4+ CD25+ CD127− regulatory T-cell (Treg) levels were significantly reduced (*p* = 0.012), indicating a shift toward a proinflammatory immune profile [22]. Similarly, Koksal et al. evaluated proinflammatory cytokines and reported significantly elevated IL-8, IL-10, and IL-17 levels in IGM patients compared with controls, whereas IL-4 and TNF-α levels did not differ significantly [35]. In another study, Ates et al. investigated the role of triggering receptor expressed on myeloid cells-1 (TREM-1), a key amplifier of inflammatory signaling that enhances IL-6, IL-8, and TNF-α production, and found significantly higher TREM-1 levels in patients with IGM. Given that TREM-1 activation has also been implicated in autoimmune diseases such as rheumatoid arthritis and systemic lupus erythematosus, these findings further support immune dysregulation as a central mechanism in IGM and suggest TREM-1 as a potential therapeutic target [36]. The presence of concomitant autoimmune diseases was evaluated in both patients and healthy volunteers in our study. A total of 13 individuals were found to have an accompanying autoimmune condition, with Hashimoto’s thyroiditis being the most frequently identified (8 patients; 61.5%). Family history of breast cancer was also assessed, and no statistically significant differences were observed between the patient and control groups with respect to the presence of autoimmune diseases or a family history of breast cancer (*p* > 0.05).

In a study including 41 patients, Cetinkaya et al. investigated the utility of the neutrophil-to-lymphocyte ratio (NLR) and platelet-to-lymphocyte ratio in predicting recurrence of idiopathic granulomatous mastitis. Both preoperative and postoperative values were evaluated, and only the preoperative NLR was found to be significantly associated with recurrent IGM (*p* = 0.024), predicting recurrence with a sensitivity of 62.5% and a specificity of 84.8% [37]. Consistent with these findings, in our study neutrophil, leukocyte, and lymphocyte counts, as well as the NLR, were significantly higher in the patient group compared with the control group in our study (*p* < 0.05).

Although the precise initiating factor remains unclear, accumulating evidence indicates that aberrant immune-mediated responses against normal breast tissue may be involved. Recent whole-exome sequencing studies have provided emerging evidence for a genetic contribution to IGM, identifying novel single nucleotide variants in immune-related genes such as NCF1, CFTR, PTPN22, HLA-DRB1, C3, and BRCA2. Notably, the NCF1 rs10614 variant—previously associated with reduced ROS production and impaired neutrophil function—suggests a potential mechanistic link between genetic susceptibility, altered oxidative balance, and chronic inflammation. Dysregulated ROS homeostasis may promote persistent immune activation and genomic instability, thereby contributing to the inflammatory microenvironment observed in GM. In parallel, variants affecting immune tolerance and antigen presentation, including PTPN22 rs2476601 and multiple HLA-DRB1 variants, support the concept of defective immune regulation and T-cell–mediated autoimmunity [38,39]. In this context, our findings of increased oxidative stress parameters and mononuclear leukocyte DNA damage further strengthen the hypothesis that IGM develops in genetically predisposed individuals through interconnected pathways involving immune dysregulation and oxidative stress. Although the identified variants are classified as benign and require functional validation, the convergence of genetic susceptibility, oxidative imbalance, and immune activation highlights a plausible integrative mechanism underlying IGM pathogenesis.

Oxidative stress has been consistently implicated in the pathogenesis of multiple autoimmune and autoinflammatory diseases—including systemic lupus erythematosus (SLE), rheumatoid arthritis, ankylosing spondylitis, inflammatory bowel disease, Hashimoto’s thyroiditis, and familial Mediterranean fever—and is particularly relevant given the frequent coexistence of IGM with autoimmune conditions such as SLE and Sjögren’s syndrome [40]. Previous studies have demonstrated increased oxidative DNA damage and impaired antioxidant defense in these conditions. For instance, Buczynska et al. reported significantly elevated levels of 8-OHdG, a marker of oxidative DNA damage, in patients with Hashimoto’s thyroiditis compared with healthy controls [41]. Similarly, Da Silva et al. highlighted that excessive production of ROS and autoantibodies in autoimmune diseases accelerates tissue damage and disease progression, while suggesting antioxidant strategies as a potential adjunctive therapeutic approach [42]. In inflammatory bowel disease, Muro et al. demonstrated significantly increased oxidative stress markers, including malondialdehyde and 8-OHdG, which correlated with disease severity, and Aslan et al. further showed increased DNA damage assessed by comet assay alongside elevated TOS and OSI values [43,44]. In rheumatoid arthritis, Kundu et al. identified markedly increased ROS production in neutrophils, which showed a strong positive correlation with inflammatory markers such as CRP [45]. Likewise, Souliotis et al. demonstrated significantly increased DNA damage parameters in patients with systemic lupus erythematosus using the comet assay technique [46]. The present study extends the current understanding of IGM by demonstrating, for the first time, the coexistence of increased oxidative stress, oxidative DNA damage, and mononuclear leukocyte injury in patients with IGM evaluated during remission and in the absence of active immunosuppressive treatment. Unlike previous studies primarily focused on inflammatory or immunological markers, our findings support a broader pathophysiological framework linking oxidative imbalance with chronic immune-mediated inflammation in IGM. These results may provide a rationale for future investigations evaluating antioxidant-based therapeutic approaches and oxidative stress–targeted biomarkers in disease monitoring and treatment response assessment.

## 5. Conclusions

This study evaluated oxidative stress and the levels of DNA damage associated with oxidative stress in IGM patients, addressing a gap in the existing literature in which oxidative stress and DNA damage have not previously been assessed together in this disease. Our findings demonstrated significantly increased total oxidant status, urinary 8-OhdG levels, OSI, and oxidative burden in patients with IGM. Furthermore, DNA damage parameters assessed by the comet assay, including tail moment and tail intensity, were markedly elevated compared with healthy controls. These results indicate that oxidative stress-induced DNA damage may play a contributory role in the pathogenesis of IGM. Importantly, all patients were evaluated during the remission phase and were not receiving active medical therapy at the time of sampling, minimizing the potential influence of treatment on oxidative stress markers and suggesting that the observed alterations more accurately reflect the underlying pathophysiology of the disease. Given the absence of a standardized treatment algorithm for IGM, identifying oxidative stress and DNA damage as potential pathogenic mechanisms may provide a rationale for exploring antioxidant-based therapeutic strategies, similar to those investigated in other autoimmune diseases. Moreover, our findings may serve as a foundation for future studies focusing on disease-specific autoantibodies, genetic susceptibility, and targeted treatment approaches, thereby contributing to improved diagnostic and therapeutic strategies for IGM.

## Figures and Tables

**Table 1:** Demographic characteristics of patient and control groups.

		Control Group (*n* = 27)				Patient Group (*n* = 28)				*p*	
		Mean ± SD/n-%			Median	Mean ± SD/n-%			Median		
Age		35.4	±	8.6	32.0	37.3	±	5.3	37.5	0.081	^m^
Age at menarche		13.7	±	1.4	14.0	12.8	±	1.1	13.0	0.006	^m^
Parity	(−)	9		33.3%		4		14.3%		0.179	^χ2^
	(+)	18		66.7%		24		85.7%			
Age at first birth		23.8	±	5.5	21.0	23.5	±	4.6	23.5	0.901	^m^
Number of births		1.8	±	0.7	2.0	2.6	±	1.1	2.0	0.055	^m^
Spontaneous abortion	(−)	26		96.3%		21		75.0%		0.025	^χ2^
	I	1		3.7%		6		21.4%			
	II	0		0.0%		1		3.6%			
Induced abortion	(−)	25		92.6%		23		82.1%		0.245	^χ2^
	I	1		3.7%		4		14.3%			
	II	1		3.7%		1		3.6%			
History of breastfeeding	(−) (+)	9 18		33.3% 66.7%		4 24		14.3% 85.7%		0.179	^χ2^
Presence of autoimmune disease	(−) (+)	20 7		74.1% 25.9%		22 6		78.6% 21.4%		0.695	^χ2^
Family history of breast cancer	(−) (+)	24 3		88.9% 11.1%		25 3		89.3% 10.7%		0.962	^χ2^
***Menopausal status*** Premenopausal Postmenopasal		24 3		88.9% 11.1%		280		100.0% 0.0%		0.111	^χ2^
Smoking status	(−) (+)	14 13		51.9% 48.1%		18 10		64.3% 35.7%		0.350	^χ2^
Oral contraceptive use	(−) (+)	26 1		96.3% 3.7%		26 2		92.9% 7.1%		1.000	^χ2^

^m^: Mann–Whitney U test; ^χ2^: chi-square test; SD: Standard Deviation.

**Table 2:** Clinical characteristics of the patients.

		Number of Patient	*n*-%
History of abscess drainage	(−)	7	25.0%
	(+)	21	75.0%
Presence of axillary lymphadenopathy	(−)	7	25.0%
	(+)	21	75.0%
Presence of breast fistula	(−)	13	46.4%
	(+)	15	53.6%
Prior antibiotic use	(−)	4	14.3%
	(+)	24	85.7%
History of systemic steroid use	(−)	16	57.1%
	(+)	12	42.9%
History of topical steroid use	(−)	18	64.3%
	(+)	10	35.7%
Biopsy method	Core needle biopsy	22	78.6%
	Excisional biopsy	5	17.8%
	Fine-needle aspiration biopsy (FNAB)	1	3.6%

**Table 3:** Comparison of hematological, biochemical, serological, and oxidative stress parameters between patient and control groups.

	Control Group (*n* = 27)				Patient Group (*n* = 28)				*p*	
	Mean ± SD			Median	Mean ± SD			Median		
WBC (×10^9^/L)	7.5	±	1.1	7.8	8.7	±	1.4	8.61	0.002	^t^
Neutrophil (×10^9^/L)	3.9	±	1.1	3.8	6.5	±	1.4	6.3	0.000	^t^
Lymphocyte (×10^9^/L)	1.8	±	0.4	1.8	2.2	±	0.7	2.2	0.027	^m^
NLR	2.04	±	0.57	2.06	3.02	±	1.1	3.05	0.007	^m^
HB (g/dL)	13.5	±	1.0	13.6	12.5	±	1.2	12.6	0.003	^t^
HCT (%)	43.3	±	2.8	44.4	40.4	±	4.2	41.1	0.008	^m^
PLT (×10^9^/L)	301.7	±	44.4	312.0	291.0	±	47.4	276.0	0.395	^t^
MCV (fL)	88.4	±	2.8	88.6	88.2	±	2.8	87.9	0.844	^t^
MCH (pg)	30.0	±	1.6	29.9	29.9	±	1.6	29.7	0.730	^t^
MCHC (g/dL)	33.6	±	1.3	33.8	33.6	±	1.2	33.7	0.821	^t^
Urea (mg/dL)	31.0	±	5.5	29.7	47.1	±	6.0	44.4	0.000	^m^
Creatinine (mg/dL)	0.78	±	0.20	0.74	1.14	±	0.32	1.28	0.000	^m^
AST (U/L)	20.1	±	6.1	19.0	21.5	±	6.4	21.5	0.418	^m^
ALT (U/L)	29.9	±	10.7	31.0	30.0	±	11.1	29.5	0.970	^t^
LDH (U/L)	157.5	±	35.9	149.0	274.8	±	35.6	267.0	0.000	^t^
Serum albumin (g/dL)	3.9	±	0.5	3.9	3.9	±	0.6	3.9	0.993	^t^
Serum sodium (mmol/L)	139.4	±	3.8	139.0	138.9	±	3.6	139.0	0.563	^t^
Serum potassium (mmol/L)	3.8	±	0.3	3.7	4.9	±	0.5	4.9	0.000	^m^
TOS (µmol H_2_O_2_ Equiv./L)	1.04	±	0.71	0.81	2.39	±	2.27	1.43	0.009	^m^
TAS (mmol Trolox Equiv./L)	0.85	±	0.32	0.91	0.89	±	0.22	0.90	0.534	^t^
Urine 8-OHdG (ng/mL)	23.62	±	19.2	25.63	36.3	±	8.5	35.0	0.024	^m^
OSI (arbitrary unit, AU)	155.2	±	168.2	116.7	284.7	±	296.5	178.3	0.028	^m^

^m^: Mann–Whitney U test, ^t^: Student’s *t* test.

**Table 4:** Tail intensity, Tail moment Analysis of control and patient groups.

		Min-Max				Median		Mean ± SD		
Tail İntensity %		0.003		-	16.58	3.45		4.84	±	5.0
Tail Moment		0.0001		-	51.74	16.51		17.16	±	14.7

	Control Group (n = 15)				Patient Group (n = 14)				*p*	
	Mean ± SD			Median	Mean ± SD			Median		
Tail İntensity %	3.15	±	4.1	1.80	6.78	±	5.3	5.26	0.029	^m^
Tail Moment	10.8	±	11.9	7.71	24.0	±	15.0	22.29	0.016	^m^

^m^: Mann–Whitney U test.

**Table 5:** Correlation analysis of DNA damage, 8-OHdG, and oxidative stress markers in patient and control groups.

		Tail Intensity %	Tail Moment	TOS	TAS	OSI
Tail Moment	r	0.64				
	*p*	0.0007				
TOS	r	0.729	0.69			
	*p*	0.000	0.000			
TAS	r	−0.511	−0.528	−0.529		
	*p*	0.005	0.003	0.003		
OSİ	r	0.735	0.734	0.961	−0.727	
	*p*	0.000	0.002	0.001	0.001	
8-OHDG	r	0.449	0.556	0.664	−0.522	0.662
	*p*	0.015	0.002	0.000	0.004	0.000

Pearson-Spearman Correlation r: correlation coefficient; *p*: *p*-value for correlation analysis.

## Data Availability

The datasets generated during this study are not publicly available for ethical reasons but are available from the corresponding author upon reasonable request.

## Abbreviations

The following abbreviations are used in this manuscript:

IGM	Idiopathic granulomatous mastitis
TOS	Total oxidant status
TAS	Total antioxidant status
OSI	Oxidative stress index
8-OhdG	8-hydroxy-2′-deoxyguanosine
DNA	Deoxyribonucleic acid
ROS	Reactive oxygen species
ROC	Receiver operating characteristic
ELISA	Enzyme-linked immunosorbent assay
WBC	White blood cell
NLR	Neutrophil-to-lymphocyte ratio
Hb	Hemoglobin
Hct	Hematocrit
NK	Natural killer cell
TREM-1	Triggering receptor expressed on myeloid cells-1
SLE	Systemic lupus erythematosus
